# Perceptions of residents and graduates regarding the theoretical-practical teaching model in orthopedics

**DOI:** 10.31744/einstein_journal/2026AO2040

**Published:** 2026-10-13

**Authors:** Marcus Vinicius Mota Garcia Moreno, Priscila Mina Falsarella, Pedro Henrique Machado Carani, Miguel José Francisco, Guilherme Francchini Santos, Guilherme Ciconelli Del Guerra, Luiz Guilherme Carvalho Hartmann, Victor Arantes Jabour, Cesar Augusto Passos Braga, Marcelo Rocha Correia da Silva, Rodrigo Gobbo Garcia, Marcos Roberto Gomes de Queiroz

**Affiliations:** 1 Hospital Cardio Pulmonar Salvador BA Brazil Hospital Cardio Pulmonar, Salvador, BA, Brazil.; 2 Hospital Israelita Albert Einstein São Paulo SP Brazil Hospital Israelita Albert Einstein, São Paulo, SP, Brazil..; 3 Hospital Israelita Albert Einstein Faculdade Israelita de Ciências da Saúde Albert Einstein São Paulo SP Brazil Faculdade Israelita de Ciências da Saúde Albert Einstein, Hospital Israelita Albert Einstein, São Paulo, SP, Brazil.

**Keywords:** Internship and residency, Orthopedics, Education, Medical Graduate, Program evaluation, Surveys and questionnaires, Brazil

## Abstract

**Objective:**

To assess residents’ and graduates’ perceptions of the theoretical-practical teaching and training model in Orthopedics and Traumatology residency programs in Bahia, Brazil; to determine whether the program meets educational expectations; and to suggest improvements.

**Methods:**

In this multicenter, cross-sectional observational study, a structured questionnaire was administered to 106 current and former orthopedic residents. It addressed the teaching model, workload, scientific training, and interpersonal relationships.

**Results:**

Of 106 respondents, 22 were excluded for incomplete responses, leaving 84. Overall, 77 (91.7%) recognized a need to improve residency training, yet 66 (78.6%) felt adequately prepared for professional practice on completion. Proposed improvements included expanding theoretical activities and lectures (36; 42.9%), strengthening practical training (40; 47.6%), and improving journal club article discussion sessions (48; 57.1%).

**Conclusion:**

Orthopedics and Traumatology residency programs in Bahia are generally perceived to meet residents’ theoretical and practical learning needs. Enhancing scientific output and interpersonal development may further improve training outcomes.

## Highlights

■ Most respondents recognized the need to improve residency training.
■ Nineteen participants perceived the orthopedic job market as offering many opportunities.
■ Overall, 78.6% of respondents felt that orthopedic residency programs adequately prepare residents for the job market.

## In Brief

Most current and former residents of Orthopedics and Traumatology programs in Bahia rated their training and performance positively, indicating satisfactory preparation for the orthopedic job market. However, participants also reported limited support for scientific research and a need to expand practical experience through subspecialty rotations, outpatient care, and trauma emergency care.

## Graphical abstract

**Figure:**
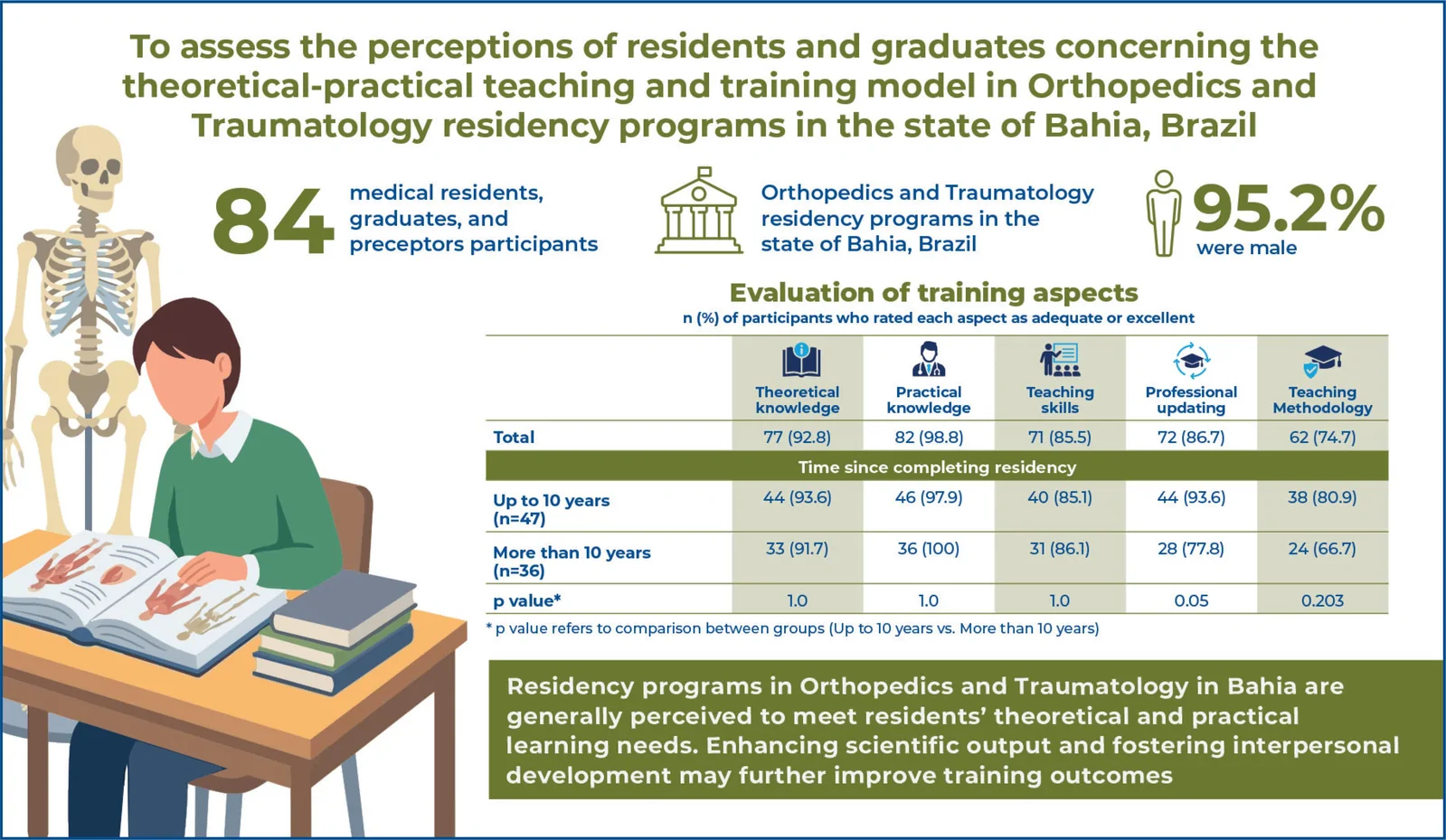

## INTRODUCTION

Medical residency, as a means of specialization in a medical field after graduation, was first proposed in 1889 by the surgeon William Halsted in the United States.^(1)^ The American Medical Association formally recognized the model in 1927. In Brazil, the first program was established in 1945 in the Department of Orthopedics at *Hospital das Clínicas, Faculdade de Medicina, Universidade de São Paulo* (USP).^(2)^

In 1967, the National Association of Medical Residents (ANMR - *Associação Nacional dos Médicos Residentes*) was established. Decree No. 80.281 of September 5, 1977,^(3)^ then recognized medical residency as the formal model for specialization in Brazil and created the Brazilian National Commission on Medical Residency (CNRM - *Comissão Nacional de Residência Médica*), regulating residency nationwide.^(3)^ Programs accredited by the Ministry of Education and Culture (MEC - *Ministério da Educação*) constitute the residency officially recognized by the state; this is one route to the Specialist Qualification Registration (REQ - *Registro de Qualificação de Especialista*) with the Regional Medical Council, permitting formal practice and legal recognition as an orthopedist. Programs accredited by the Brazilian Society of Orthopedics and Traumatology (SBOT - *Sociedade Brasileira de Ortopedia e Traumatologia*), by contrast, are evaluated by the specialty's scientific body for technical quality, case volume, educational structure, and practical training, qualifying graduates to sit the Examination for the Title of Specialist in Orthopedics and Traumatology (TEOT), but without legal validity for RQE on their own, except after the TEOT is obtained.

In Bahia, Orthopedics and Traumatology began to gain prominence in the 1940s. In 1990, the first such residency program in the state to be recognized by the SBOT was established. Named the Integrated Residency Program in Orthopedics and Traumatology of Bahia (RIBOT - *Residência Integrada Baiana de Ortopedia e Traumatologia*), it arose from a partnership among *Hospital Santa Isabel, Hospital Martagão Gesteira*, and *Hospital COT*. Several other programs followed, notably at *Hospital São Rafael, Hospital COT, Hospital Manoel Victorino, Hospital do Subúrbio*, and *Hospital Santo Antônio of Obras Sociais Irmã Dulce*.^(4)^

The health education model in medical residency is a system requiring structured pedagogical approaches to train specialists. It encompasses scientific foundations, curriculum design, theoretical-practical methodologies, and institutional frameworks.^(5)^ Interaction among health professionals from different disciplines and with patients in the controlled environment of training is also essential, forming the basis for relationships that continue throughout professional practice. When functioning effectively, these factors strengthen the teaching-learning dynamic.^(6)^

The rising number of new medical schools—and growing concern about the quality of education they provide ^(7)^—has increased the importance of residency training in qualifying health professionals for the market. Residency programs aim to ensure comprehensive, specialized learning, more effective health care, and better integration among residents, the care team, and patients.^(8–10)^

## OBJECTIVE

To assess current residents’ and graduates’ perceptions of the theoretical-practical teaching and training model in Orthopedics and Traumatology at reference centers in Bahia, Brazil; to determine whether the program meets their training and learning expectations; and to propose improvements for Orthopedics and Traumatology education.

## METHODS

This multicenter, cross-sectional observational study was approved by the Human Research Ethics Committee (*Centro de Pesquisas Gonçalo Moniz* - Fiocruz/BA - CAAE:54981721.4.3004.0040; #5.478.014; and *Hospital Israelita Albert Einstein* CAAE: 54981721.4.0000.0071; # 6.014.052). All participants signed an informed consent form.

### Research participants

Participants were active orthopedic residents (in training), graduates of orthopedic residency (physicians who completed training, whether or not they train new residents), and preceptors (graduates who train new residents). Non-resident participants were professionals currently working in orthopedic residency programs in Bahia, regardless of where they trained.

Data were collected with a digital questionnaire developed by the researchers (Table 1S, Supplementary Material) and administered through the REDCap platform.^(11)^ Participants rated the residency program on a Likert-type scale: poor, fair, good, very good, and excellent. For analysis, "good," "very good," and "excellent" were classified as satisfactory, and "poor" and "fair" as unsatisfactory.

Inclusion criteria were active orthopedic residents with at least nine months of training; graduates of orthopedic residency or of earlier tutorial-based programs (before formal residency regulation in 1990); and current preceptors training orthopedics and traumatology residents in Bahia at programs accredited by the Brazilian MEC, the SBOT, or both. Participants were included only if they completed the questionnaire and consented. The exclusion criterion was an incomplete questionnaire.

Descriptive analyses of the variables were based on absolute frequencies and percentages for categorical variables, as well as summary measures. Associations between participants’ training and qualification characteristics and their post-residency perspectives were investigated using Student's *t*-tests or Mann-Whitney tests for numerical variables, and *χ*^2^ or Fisher's exact tests for categorical variables, based on the distributions observed after data collection. Analyses were performed using IBM SPSS Statistics for Windows software, version 24.0. Analyses used IBM SPSS Statistics for Windows, version 24.0 (IBM Corp., Armonk, NY, USA).^(12)^

## RESULTS

The database contained 106 responses from residents, graduates, and preceptors; 22 were excluded for incomplete information, yielding a final sample of 84 participants.

Participants’ ages ranged from 26 to 67 years (mean, 41.9 years; SD, 8.9). Of these, 80 (95.2%) were male and four (4.8%) female (Table 1).

**Table 1:** General characteristics of study participants

Variable		n (%)
Sex		
	Male	80 (95.2)
	Female	4 (4.8)
Age (years)		
	Mean (SD)	41.9 (8.9)
	Median (Q1; Q3)	42 (34; 47)
	Minimum; maximum	26; 67
Medical school		
	Public	22 (26.2)
	Private	62 (73.8)
Residency program		
	Public	33 (39.3)
	Private	51 (60.7)
Program accreditation		
	MEC	8 (9.5)
	SBOT	44 (52.4)
	Both	32 (38.1)
Status		
	Current resident	9 (10.7)
	Graduate	75 (89.3)
Preceptor		
	No/not answered	41 (48.8)
	Yes	43 (51.2)
Years since residency completion		
	Mean (SD)	11.8 (8.9)
	Median (Q1; Q3)	9.5 (3.0; 19.0)
	Minimum; maximum	0; 40
	Number of respondents	74
Subspecialty area		
	Trauma	50 (59.5)
	Foot and ankle	20 (23.8)
	Knee	17 (20.2)
	Pain management	14 (16.7)
	Shoulder and elbow	12 (14.3)
	Hip	11 (13.1)
	Sports medicine	10 (11.9)
	Pediatrics	7 (8.3)
	Tumor	6 (7.1)
	Spine	5 (6.0)
	Hand	3 (3.6)

SD: standard deviation; Q1: first quartile; Q3: third quartile; MEC: Brazilian Ministry of Education and Culture; SBOT: Brazilian Society of Orthopedics and Traumatology.

Regarding medical school, 22 participants (26.2%) attended public institutions and 62 (73.8%) private institutions. For residency, 33 (39.3%) trained in public hospitals and 51 (60.7%) in private hospitals. By program accreditation, eight (9.5%) were affiliated with MEC-accredited programs, 44 (52.4%) with SBOT-accredited programs, and 32 (38.1%) with both (Table 1).

Among participants, 75 (89.3%) were graduates of Orthopedics and Traumatology residency programs, with time since completion ranging from less than 1 year to 40 years (median, 9.5 years; IQR, 3 to 19). Nine (10.7%) were current residents — one PGY-1, three PGY-2, and five PGY-3. In addition, 43 (51.2%) were preceptors actively training residents in Bahia at the time of the survey, regardless of where they trained.

By subspecialty, 50 participants (59.5%) worked in trauma, 20 (23.8%) in foot and ankle, 17 (20.2%) in knee, 14 (16.7%) in pain management, 12 (14.3%) in shoulder and elbow, 11 (13.1%) in hip, 10 (11.9%) in sports medicine, seven (8.3%) in pediatrics, six (7.1%) in tumors, five (6.0%) in spine, and three (3.6%) in hand surgery.

The overall evaluation of the medical residency program, including both theoretical and practical components, as well as the evaluation of the preceptorship, are described in tables 2 to 4.

**Table 2:** Satisfaction with the residency program

	Total (n=83) – n (%)	Time since completing residency		p value*
		Up to 10 years (n=47) – n (%)	>10 years (n=36) – n (%)	
Preceptor competence and training	77 (92.8)	46 (97.9)	31 (86.1)	0.081
Institutional tradition in scientific research	56 (67.5)	34 (72.3)	22 (61.1)	0.347
Stipend/remuneration	38 (45.8)	22 (46.8)	16 (44.4)	1.0
Workload	65 (78.3)	34 (72.3)	31 (86.1)	0.181
Theoretical training	67 (80.7)	39 (83.0)	28 (77.8)	0.585
Practical training	75 (90.4)	44 (93.6)	31 (86.1)	0.284
Opportunity for rotation across subspecialties	67 (80.7)	40 (85.1)	27 (75.0)	0.274
Job market placement	72 (86.7)	42 (89.4)	30 (83.3)	0.520

* Fisher's Exact Test. 83 respondents.

**Table 3:** Satisfaction with the theoretical and practical components of the residency program

		Total (n=83) – n (%)	Time since completing residency		p value*
			Up to 10 years (n=47) – n (%)	More than 10 years (n=36) – n (%)	
Theoretical program					
	Content	74 (89.2)	42 (89.4)	32 (88.9)	1.0
	Actual learning	78 (93.4)	44 (93.4)	34 (94.4)	1.0
	Preparation for clinical practice	79 (95.2)	46 (97.9)	33 (91.7)	0.312
	Preparation for the OTBE	71 (85.5)	39 (83)	32 (88.9)	0.539
	Frequency of scientific meetings	12 (14.5)	8 (19)	4 (11.8)	0.531
Practical program					
	Content	80 (96.4)	46 (97.9)	34 (94.4)	0.576
	Actual learning	75 (90.4)	44 (93.6)	31 (86.1)	0.284
	Preparation for clinical practice	80 (96.4)	46 (97.9)	34 (94.4)	0.576
	Preparation for OTBE	70 (84.3)	37 (78.7)	33 (91.7)	0.135

* Fisher's Exact Test. 83 respondents.

OTBE: Orthopedics and Traumatology Board Examination.

**Table 4:** Satisfaction with preceptorship

	Total (n=83)	Time since completing residency		p value*
		Up to 10 years (n=47)	More than 10 years (n=36)	
Theoretical knowledge	77 (92.8)	44 (93.6)	33 (91.7)	1.0
Practical knowledge	82 (98.8)	46 (97.9)	36 (100)	1.0
Teaching skills	71 (85.5)	40 (85.1)	31 (86.1)	1.0
Professional updating	72 (86.7)	44 (93.6)	28 (77.8)	0.05
Teaching Methodology	62 (74.7)	38 (80.9)	24 (66.7)	0.203

* Fisher's Exact Test. 83 respondents.

Regarding post-residency perspectives, 19 participants (22.6%) viewed the orthopedic job market as still offering many opportunities, and 21 (25.0%) reported receiving guidance on the profession and job market during residency. Looking ahead, 58 (69.0%) planned to remain involved with residency programs as preceptors, and 50 (59.5%) intended to pursue further academic qualifications, such as a master's or doctoral degree.

Regarding the training model they attended or are attending, 77 participants (91.7%) believed improvements are needed in orthopedic resident training. However, 66 (78.6%) felt they graduated, or will graduate, adequately prepared for the job market. Asked about the main actions needed, 36 (42.9%) cited enhanced theoretical activities and lectures, 40 (47.6%) improved practical training, and 48 (57.1%) better article discussion sessions. Others recommended complementary internships (33; 39.3%), more free time for independent study (29; 34.5%), improved rotations at other hospitals (23; 27.4%), more time in trauma emergency care (19; 22.6%), and greater outpatient clinic experience (15; 17.9%).

## DISCUSSION

Few studies have evaluated medical residency education or the experiences and outcomes of resident training. Through a questionnaire administered to orthopedists currently working in Bahia, this study assessed the theoretical-practical teaching and training model used in orthopedic residency programs in the state and explored individual and professional variables potentially associated with these perceptions.^(13)^ Such evaluation is especially relevant given the recent increase in orthopedic residency programs nationwide.

Most participants were male (95.2%), consistent with national 2022 data showing that 92.6% of orthopedic residents in Brazil were men.^(14)^ This alignment reinforces the external validity of our findings.

In evaluating training, 92.8% of participants were satisfied with the competence and training of the preceptor faculty, 90.4% with practical training, and 86.7% with integration into the job market. Lower satisfaction was reported for the program's scientific research tradition (67.5%) and resident stipend/remuneration (45.8%).

Preparation for the Orthopedics and Traumatology Board Examination (OTBE) is an important aspect of training. Most residents reported adequate theoretical and practical content and preparation (85.5% and 84.3%, respectively). No statistically significant difference was observed between subgroups with up to 10 years and more than 10 years since completing residency.

With the growing number and diversity of residency programs, concerns have arisen about hospital infrastructure and resources for effective learning. Most participants were satisfied with the physical space, materials, and facilities provided, though notable dissatisfaction centered on absent or inadequate libraries and study centers, which are essential scientific support for residents’ academic activities.

A common concern when choosing a program is the availability of a broad range of subspecialties. Among the programs evaluated, the large majority (77.4%) offered comprehensive exposure to all major areas; the most frequently reported gaps were in pain management and sports medicine.

Most participants also rated their study routines and residency programs positively. However, 21.4% of training centers reported no annual scientific output, and only 15.8% were satisfied with support for research activities, such as scientific meetings.

The study also assessed perceptions of the job market for orthopedic surgeons in Brazil. Only 22% of respondents perceived favorable opportunities and sufficient room for career development.

This study has several limitations. First, most responses came from graduates, with relatively few from current residents. Second, it was limited to programs in Bahia, which may restrict generalizability to other regions of Brazil; nonetheless, the findings encourage similar studies elsewhere to better characterize orthopedic—and other specialty—training nationwide. Third, the number of residency graduates and practicing orthopedists in Bahia was unknown, preventing calculation of the sample's representativeness. Finally, 22 participants were excluded for incomplete online questionnaires, and no clear pattern of non-response was identified.

## CONCLUSION

Most current and former residents of Orthopedics and Traumatology programs in Bahia rated their training and residency performance positively, indicating satisfactory preparation for the orthopedic job market. However, participants also reported limited support for scientific research and a need to expand practical experience through subspecialty rotations, outpatient clinic activities, and trauma emergency care.

## Data Availability

The underlying content is contained within the manuscript.
