# Supplementary material for: Perceptions of residents and graduates regarding the theoretical-practical teaching model in orthopedics
Source: Einstein (Sao Paulo). 2026 Oct 13;24:eAO2040. doi: 10.31744/einstein_journal/2026AO2040 (PMC13682775; doi:10.31744/einstein_journal/2026AO2040)
Supplement: Supplementary file 1 [file 2317-6385-eins-24-eAO2040-Suppl01.pdf]

SUPPLEMENTARY MATERIAL

Perceptions of residents and graduates regarding the theoretical-practical teaching model in orthopedics

Marcus Vinicius Mota Garcia Moreno, Priscila Mina Falsarella, Pedro Henrique Machado Carani, Miguel José Francisco Neto, Guilherme Francchini Santos, Guilherme Ciconelli Del Guerra, Luiz Guilherme Carvalho Hartmann, Victor Arantes Jabour, Cesar Augusto Passos Braga, Marcelo Rocha Correia da Silva, Rodrigo Gobbo Garcia, Marcos Roberto Gomes de Queiroz

DOI: 10.31744/einstein_journal/2026AO2040

Table 1S. Evaluation of residents' and graduates' perception of the teaching model in orthopedics

**Identification:**
Gender: ( ) Male ( ) Female ( ) Other ( ) Prefer not to say
Age:
College/University of graduation: ( ) Public ( ) Private
Residency Program: ( ) Public ( ) Private
Accreditation: ( ) MEC ( ) SBOT ( ) Both
Resident: ( ) Yes ( ) No (Graduate) Year: ( ) R1 ( ) R2 ( ) R3
Preceptor: ( ) Yes ( ) No
Time since residency completion: ( ) Years
Area of expertise: (you can select more than one option)
( ) Trauma ( ) Spine ( ) Shoulder and Elbow ( ) Hand ( ) Tumor ( ) Pediatric
( ) Hip ( ) Knee ( ) Foot and Ankle ( ) Sports ( ) Pain
**Reason for Choosing the Residency Program:**
Location: ( ) Capital ( ) Interior
Type: ( ) Public ( ) Private
Capacity and Training of Clinical Staff – Preceptors: ( ) Yes ( ) No
Scientific Tradition of the Service: ( ) Yes ( ) No
Scholarship / Remuneration: ( ) Yes ( ) No
Workload: ( ) Yes ( ) No
Theoretical Training: ( ) Yes ( ) No
Practical Training: ( ) Yes ( ) No
Possibility of rotation in different areas: ( ) Yes ( ) No
Possibility of internship in the area of expertise: ( ) Yes ( ) No
Job Market Integration: ( ) Yes ( ) No
**Selection process:**
Multiple-choice test ( ) Yes ( ) No
Subjective test ( ) Yes ( ) No
Curriculum analysis ( ) Yes ( ) No
Interview ( ) Yes ( ) No
Psychological test ( ) Yes ( ) No
**Theoretical program:**
Content ( ) poor ( ) fair ( ) good ( ) excellent
Actual learning ( ) poor ( ) fair ( ) good ( ) excellent
Training for practical life ( ) poor ( ) fair ( ) good ( ) excellent
Preparation for TEOT OTBE( ) poor ( ) fair ( ) good ( ) excellent
Workload ( ) poor ( ) fair ( ) good ( ) excellent
Any form of active learning? ( ) Team-Based Learning ( ) PBL ( ) Discussion
Scientific Meetings ( ) Yes ( ) No
If yes, frequency of meetings ( ) daily ( ) weekly ( ) monthly
**Practical program:**
Content ( ) poor ( ) average ( ) good ( ) excellent
Actual learning ( ) poor ( ) average ( ) good ( ) excellent
Training for practical life ( ) poor ( ) average ( ) good ( ) excellent
Preparation for TEOT ( ) poor ( ) average ( ) good ( ) excellent
Workload ( ) poor ( ) average ( ) good ( ) excellent

Hospital structure – residency:
Equipment ( ) poor ( ) average ( ) good ( ) excellent
Facilities ( ) poor ( ) average ( ) good ( ) excellent
Study Center ( ) absent ( ) poor ( ) average ( ) good ( ) excellent
Library ( ) absent ( ) poor ( ) average ( ) good ( ) excellent
Diagnostics (exams etc.) ( ) absent ( ) poor ( ) average ( ) good ( ) excellent
SBOT Bibliography ( ) absent ( ) poor ( ) average ( ) good ( ) excellent

Preceptorship:
Theoretical knowledge ( ) poor ( ) average ( ) good ( ) excellent
Practical knowledge ( ) poor ( ) average ( ) good ( ) excellent
Teaching skills ( ) poor ( ) average ( ) good ( ) excellent
Continuing education ( ) poor ( ) average ( ) good ( ) excellent
Skills ( ) poor ( ) average ( ) good ( ) excellent
Teaching methodology ( ) poor ( ) average ( ) good ( ) excellent
Does the teaching staff practice Continuing Education? ( ) yes ( ) no

Periodic assessments – tests:
Theoretical ( ) nonexistent ( ) rare ( ) regular ( ) excessive
Practical ( ) nonexistent ( ) rare ( ) regular ( ) excessive
Skills ( ) nonexistent ( ) rare ( ) regular ( ) excessive
Videos ( ) nonexistent ( ) rare ( ) regular ( ) excessive
Does the service offer all areas of orthopedic care? ( ) Yes ( ) No
Which areas of care are not offered by the service? (You can select more than one option)
( ) Trauma ( ) Spine ( ) Shoulder and Elbow ( ) Hand ( ) Tumor ( ) Pediatric ( ) Hip ( ) Knee ( ) Foot and Ankle ( ) Sports ( ) Pain
Do you have a regular study routine? ( ) Yes ( ) No
How many hours a day do you study? ( )<1h ( ) 1-2h ( ) 2-3h ( ) >3h
Rate your residency experience (0-10):
( ) 0 ( ) 1 ( ) 2 ( ) 3 ( ) 4 ( ) 5
( ) 6 ( ) 7 ( ) 8 ( ) 9 ( ) 10
Rate your performance in residency (0-10):
( ) 0 ( ) 1 ( ) 2 ( ) 3 ( ) 4 ( ) 5
( ) 6 ( ) 7 ( ) 8 ( ) 9 ( ) 10
Weekly workload dedicated to residency work:
( )<30h ( ) 30-60h ( )>60h
Weekly workload dedicated to work outside residency:
( )<12h ( ) 12-24h ( ) 24-48h ( ) >48h
Scientific production and publication regarding residency services (annual):
( ) nonexistent ( ) 1-5 ( ) 6-10 ( ) >10
Is there/was there an incentive for scientific production? ( ) yes ( ) no
If yes, what type: ( ) preparation of panels-posters for congresses ( ) preparation of scientific papers ( ) articles
Was a scientific period made available? ( ) yes ( ) no

Regarding personal relationships:
Regarding relationships with your residency peers:
How would you rate your relationship with your residency peers (in general):
( ) poor ( ) fair ( ) good ( ) excellent
How would you rate your relationship with your residency peers (from the same period):
( ) poor ( ) fair ( ) good ( ) excellent
How would you rate your relationship with your residency peers (older):
( ) poor ( ) fair ( ) good ( ) excellent
How would you rate your relationship with your residency peers (newer):
( ) poor ( ) fair ( ) good ( ) excellent
Do you believe you need to improve your relationship with your residency peers?
( ) yes ( ) no ( ) indifferent

Regarding your relationship with your residency preceptors:
How would you rate your relationship with your residency preceptors:
( ) poor ( ) fair ( ) good ( ) excellent
Do you have more affinity with a preceptor in your area of interest:
( ) yes ( ) no ( ) indifferent
If there is affinity with a particular preceptor, did this influence your choice of area of interest:
( ) yes ( ) no ( ) indifferent
Do you believe you need to improve your relationship with your preceptors?
( ) yes ( ) no ( ) indifferent

Regarding the relationship with the healthcare team:

In your relationship with other members of the healthcare team, do you greet them with due respect and attention?

( ) yes ( ) no ( ) I don't know

Do you have a routine of identifying yourself and communicating effectively and directly?

( ) yes ( ) no ( ) I don't know

Do you think you maintain a good relationship and listen to the demands they bring?

( ) yes ( ) no ( ) I don't know

Do you have appropriate attitudes and expressions when dealing with other non-medical professionals?

( ) Yes ( ) No ( ) I don't know

Do you have a greater affinity for work with any specific profession among non-medical professionals?

( ) Nurses ( ) Nursing Technicians ( ) Physiotherapist ( ) Social Worker ( ) Administrative Sector ( ) General Services ( ) Others

Do you have greater difficulty in your work relationship with any specific profession among non-medical professionals?

( ) Nurses ( ) Nursing Technicians ( ) Physiotherapist ( ) Social Worker ( ) Administrative Sector ( ) General Services ( ) Others

Do you believe you need to improve your relationship with other non-medical professionals?

( ) Yes ( ) No ( ) Indifferent

Regarding patient relationships:

In your relationship with patients, do you greet them with due respect and attention?

( ) yes ( ) no ( ) I don't know

Do you routinely identify yourself and speak in an ethical and direct manner?

( ) yes ( ) no ( ) I don't know

Do you think they maintain a good relationship and do you listen to the demands they bring?

( ) yes ( ) no ( ) I don't know

Do you have appropriate attitudes and expressions when dealing with patient companions and caregivers?

( ) Yes ( ) No ( ) I don't know

Do you have a greater affinity in your relationship with a particular socioeconomic class of patients?

( ) Low ( ) Medium ( ) High

Do you have greater difficulty in your relationship with a particular socioeconomic class of patients?

( ) Low ( ) Medium ( ) High

Do you believe you need to improve your relationship with patients and their companions?

( ) Yes ( ) No ( ) Indifferent

Post-residency:

Do you imagine that the job market for doctors, in general, in our country is still broad and with many opportunities?

( ) yes ( ) no ( ) I don't know ( ) depends on the region

Do you imagine that the job market for orthopedists, in general, in our country is still broad and with many opportunities?

( ) yes ( ) no ( ) I don't know ( ) depends on the region

Do you imagine that the job market for orthopedists, with a specific area of expertise, in our country is better than that of general practitioners and with more opportunities?

( ) yes ( ) no ( ) I don't know ( ) depends on the region

Do you intend to pursue training in a specific area of orthopedics?

( ) yes ( ) no

Desired area of practice: (you can select more than one option)

( ) Trauma ( ) Spine ( ) Shoulder and Elbow ( ) Hand ( ) Tumor ( ) Childhood Pediatrics

( ) Hip ( ) Knee ( ) Foot and Ankle ( ) Sports ( ) Pain ( ) N/A

Do you intend to continue being affiliated with a residency program as a preceptor?

( ) yes ( ) no ( ) I don't know

Do you intend to pursue further academic training (Master's/PhD)?

( ) yes ( ) no ( ) I don't know

Regarding the medical residency training model you participated in or are participating in:

Is there a need to improve the training of residents?

( ) yes ( ) no ( ) I don't know

Do you consider that you left or will leave your orthopedic medical residency ready for the job market?

( ) yes ( ) no ( ) I don't know

What is the main action to improve residency in orthopedics? Focus on increased dedication or specific workload? – you can choose more than one option:

( ) theoretical activity – classes ( ) practical activity ( ) discussion of articles

( ) complementary internships ( ) more free time for study

( ) rotations in other hospitals ( ) more time in the trauma emergency room

( ) more time spent in the office
